# The Study of Electrical and Electrochemical Properties of Magnesium Ion Conducting CS: PVA Based Polymer Blend Electrolytes: Role of Lattice Energy of Magnesium Salts on EDLC Performance

**DOI:** 10.3390/molecules25194503

**Published:** 2020-10-01

**Authors:** Shujahadeen B. Aziz, Mohamad A. Brza, Elham M. A. Dannoun, Muhamad H. Hamsan, Jihad M. Hadi, Mohd F. Z. Kadir, Rebar T. Abdulwahid

**Affiliations:** 1 Advanced Polymeric Materials Research Lab., Department of Physics, College of Science, University of Sulaimani, Qlyasan Street, Kurdistan Regional Government-Iraq, Sulaimani 46001, Iraq; 2 Department of Civil Engineering, College of Engineering, Komar University of Science and Technology, Kurdistan Regional Government, Sulaimani 46001, Iraq; 3 Department of Manufacturing and Materials Engineering, Faculty of Engineering, International Islamic University of Malaysia, Kuala Lumpur 53100, Malaysia; mohamad.brza@gmail.com; 4 Associate Director of General Science Department, Woman Campus, Prince Sultan University, P.O. Box 66833, Riyadh 11586, Saudi Arabia; elhamdannoun1977@gmail.com; 5 Institute for Advanced Studies, University of Malaya, Kuala Lumpur 50603, Malaysia; hafizhamsan93@gmail.com; 6 College of Engineering, Tishk International University, Kurdistan Regional Government, Sulaimani 46001, Iraq; jihad.chemist@gmail.com; 7 Centre for Foundation Studies in Science, University of Malaya, Kuala Lumpur 50603, Malaysia; mfzkadir@um.edu.my; 8 Department of Physics, College of Education, University of Sulaimani, Old Campus, Kurdistan Regional Government-Iraq, Sulaimani 46001, Iraq; rebar.abdulwahid@univsul.edu.iq

**Keywords:** polymer blends, magnesium salt, impedance, dielectric properties, LSV and TNM, CV study, Salt lattice energy, electrochemical double-layer capacitor

## Abstract

Plasticized magnesium ion conducting polymer blend electrolytes based on chitosan (CS): polyvinyl alcohol (PVA) was synthesized with a casting technique. The source of ions is magnesium triflate Mg(CF_3_SO_3_)_2_, and glycerol was used as a plasticizer. The electrical and electrochemical characteristics were examined. The outcome from X-ray diffraction (XRD) examination illustrates that the electrolyte with highest conductivity exhibits the minimum degree of crystallinity. The study of the dielectric relaxation has shown that the peak appearance obeys the non-Debye type of relaxation process. An enhancement in conductivity of ions of the electrolyte system was achieved by insertion of glycerol. The total conductivity is essentially ascribed to ions instead of electrons. The maximum DC ionic conductivity was measured to be 1.016 × 10^−5^ S cm^−1^ when 42 wt.% of plasticizer was added. Potential stability of the highest conducting electrolyte was found to be 2.4 V. The cyclic voltammetry (CV) response shows the behavior of the capacitor is non-Faradaic where no redox peaks appear. The shape of the CV response and EDLC specific capacitance are influenced by the scan rate. The specific capacitance values were 7.41 F/g and 32.69 F/g at 100 mV/s and 10 mV/s, respectively. Finally, the electrolyte with maximum conductivity value is obtained and used as electrodes separator in the electrochemical double-layer capacitor (EDLC) applications. The role of lattice energy of magnesium salts in energy storage performance is discussed in detail.

## 1. Introduction

The solid polymer electrolytes (SPEs) were applied for lithium batteries since 1979 [1]. Over decades, it has become one of the main components in electrochemical devices, such as proton batteries and electrochemical double-layer capacitor (EDLC). These liquid electrolytes show a good performance in a few energy devices, but they evaporate easily and also have detrimental impact on internal components of the devices, for instance corrosion and leaking problems [2,3,4]. Based on these considerations, researchers have found that SPEs are a better candidate to be an alternative to the liquid electrolytes. Moreover, safety, long shelf life, and simple fabrication process are features of SPEs [5]. To enhance the mechanical strength, thermal stability as well as increasing the sites availability for ion hopping of SPEs, it is much better to blend two or more different polymers [6,7].

Now, the role of natural polymers cannot be ignored as they possess high quality properties, such as biodegradability that fulfill the requirements of green chemistry. Chitosan (CS) and poly (vinyl alcohol) (PVA) are two common examples of natural polymer that have been extensively investigated in the applications of energy storage devices [8,9,10]. CS consists mainly of a linear polysaccharide that is linked by-(1–4)-linked-d-glucosamine and N-acetyl-d-glucosamine. To obtain this polymer, the deacetylation process of chitin has to be carried out. In comparison with other polymers, CS is characterized by biodegradability, biocompatibility, low toxicity as well as affordability properties [11,12]. Similarly, PVA is a nontoxic polymer characterized by enrichment with polar oxygen atom within the vinyl alcohol groups, which facilitates complexation with cations, forming unique polymer electrolyte complexes [13,14]. It also possesses relatively high dielectric strength, high chemical stability, reasonable charge storage capacity, dopant-dependent electric, optical properties, and abrasion resistance [15]. These two types of polymers invade the literatures [16,17,18].

In an attempt to enhance the ionic conductivity of SPEs, salt addition into the electrolyte has been introduced because it is critical to be implemented in electrochemical storage devices. However, the process of salt addition is not free from problems. For example; lithium-based salts show an overall good performance with non-biodegradability which has a detrimental impact on the environment [19]. Nowadays, the H^+^, Mg^2+^, and NH_4_^+^ ions are considered as the alternatives for Li^+^ ion in SPEs [2,20]. For example, the blend of PEO/EmimBF_4_ has shown an increase in ionic conductivity with the addition of magnesium triflate (Mg(CF_3_SO_3_)_2_) salt up to 9.4 × 10^−5^ S cm^−1^ at room temperature [21]. Sarangika et al. [22] also documented a relatively high ionic conductivity for the poly(ethylene oxide) (PEO) film of 1.19 × 10^−4^ S cm^−1^ with the addition of Mg(CF_3_SO_3_)_2_ salt at room temperature. The advantages of the addition of Mg(CF_3_SO_3_)_2_ salt are cheapness and that it can be grasped at open air and abundance in nature [23]. Guan et al. [24] believed that the divalent Mg^2+^ ion is strongly attracted by the lone pair electrons of oxygen atoms whereas CF_3_SO_3_^−^ anion is characterized by delocalized negative charges across the sulfonate group, which is stabilized due to the resonance stabilization, which creates this triflate group as an excellent leaving group. This behavior facilitates salt dissolution within polymer bodies, thus enhancing the ionic mobility [25]. In another study, the impact of glycerol as a plasticizer on ionic conductivity of starch-Mg(C_2_H_3_O_2_)_2_ system was carried out and it was demonstrated that the glycerol has the ability to accelerate the salt dissociation [26]. Glycerol weakens the columbic force between cations and anions. As a consequence, ion concentration can be increased with the increasing of salt dissociation, which is desired for improving the conductivity. Mattos et al. [27] achieved a development of conductivity of ions of the electrolyte systems from 10^−7^ S cm^−1^ to 10^−5^ S cm^−1^ as a result of glycerol addition.

Herein, the electrochemical double-layer capacitor (EDLC) is one of the energy storage devices that appear as strong alternatives for the conventional batteries. Researchers have found that EDLC shows relatively high durability and power density compared to other supercapacitors (SCs) [28,29,30]. The ease and cheapness of fabrication of EDLC is an interesting feature of this device. This is due to utilization of few materials in the fabrication of EDLC, such as carbon nanotubes [31], graphite, [32] and activated carbon [33]. Nowadays, activated carbon is found to be utilized that have better performance than other materials owing to reasonable chemical durability, big surface area, and also good electronic conductivity [34]. In dealing with this material, the mechanism of energy storage in EDLC is explained that on the basis of the non-Faradaic mechanism whereby a double layer at the interfacial area is formed as a result of ion accumulation [35]. In other words, no electron transfer (i.e., Non-Faradaic process) takes place at the surface of the electrodes and the electrolytes. Instead, there is merely a buildup of charge.

In this present work, 40 w.t.% of Mg(CF_3_SO_3_)_2_ salt with various quantities of glycerol were mixed with the CS/PVA blend to prepare magnesium ion conducting polymer blend electrolytes. The impact of glycerol plasticizer on the ionic conductivity based on the electrical properties via electrical impedance spectroscopy was explored at room temperature. In the analysis of the data points, dielectric properties of the electrolytes are discussed. The relatively high conducting electrolyte is utilized in the fabrication of EDLC.

## 2. Experimental

### 2.1. Materials and Polymer Electrolyte Preparation

All chemical materials were procured from Sigma-Aldrich and directly employed with no further purification. Two main raw materials used at the preparation of glycerolized the CS:PVA:Mg(CF_3_SO_3_)_2_ blend electrolyte systems are; chitosan (CS), and polyvinyl Alcohol (PVA) with the average molecular weights of 310,000–375,000 g·moL^−1^, and 35,000 g·moL^−1^, respectively. Other raw materials are magnesium triflate Mg(CF_3_SO_3_)_2_, acetic acid (CH_3_COOH), and glycerol (C_3_H_8_O_3_) where involved in solution casting technique. The preparation of the blend comprised a dissolution of 0.5 g of CS in 30 mL of the solution of 1% acetic acid, which was then stirred for several hours at room temperature. Meanwhile, dissolution of 0.5 g in PVA in 30 mL of distilled water at 80 °C was performed with stirring for several hours. Afterwards, this solution was cooled down to room temperature. The two separate solutions (CS and PVA) were mixed and stirred continuously under magnetic stirrer condition to prepare PVA:CS polymer blends. Subsequently, 40 wt.% (0.666) of magnesium triflate Mg(CF_3_SO_3_)_2_ salt was added to the CS:PVA blended solutions, and stirred until the homogenous mixture was gained. Then, glycerol plasticizer separately with various concentrations was added to the electrolyte systems. Finally, the CS:PVA:Mg(CF_3_SO_3_)_2_ systems were coded as CSPVMG1, CSPVMG2, and CSPVMG3 with the incorporation of 14, 28, and 42 wt.% of glycerol, respectively. To reach dryness, each of the prepared solutions was poured into dry and clean labeled Petri dishes and left untouched to evaporate leisurely at ambient temperature. Table 1 displays the constitution of the solid polymer electrolyte film samples.

### 2.2. Fabrication of the EDLC

The construction of the electrochemical double-layer capacitor involves four successive simple steps. The 1st step comprises dry mixing process, in which 81.25% activated carbon (AC) and 6.25% carbon black (CB) are mixed using planetary ball miller at 500 r/min for 20 min. The 2nd step is dissolution process that AC-CB powder is dissolved in a solution of 15 mL N-methyl pyrrolidone (NMP) in the presence of 12.5% of polyvinylidene fluoride (PVdF) as a binder until appearance of thick black solution. The 3rd step is coating process. The obtained black solution can be coated on an aluminum foil via doctor blade with thickness of 0.25 mm. The last step is drying process. Subsequently, the coated aluminum foils are dried in a pre-heated oven at 60 °C for few hours. The dried electrodes are cut into a small specified circle shape with a geometric area of 2.01 cm^2^ and stored in a desiccator prior to characterization processes. The arrangement of the EDLC design is AC| best conducting sample |AC. In this design, CR2032 coin cell is mounted in a Teflon case.

### 2.3. Electrical and Electrochemical Characterization

#### 2.3.1. Impedance and Electrical Study

The LCR meter (HIOKI 3531 Z Hi-tester, Nagano, Japan) impedance analyzer was used in the electrical impedance measurements of the synthesized samples in the range of frequencies between 50 Hz and 5 MHz. In the measurement process, the free-standing films were cut into small discs with diameter of 2 cm. The CSPVMG electrolyte samples were sandwiched between two stainless-steel electrodes as blocking electrodes for with a great pressure in order to reach enough contact. The Nyquist plot of real (*Zr*) and imaginary (*Zi*) parts of complex impedance (Z*) were obtained. Finally, in the analysis process, the bulk resistance (*R_b_*) was taken from extrapolating the spike to the (*Zr-axis*) at the relatively high intercept of a semicircle for each sample.

#### 2.3.2. Transference Number Analysis (TNM)

From the analysis of the TNM, the main charge carrier species in the electrolyte was determined using V&A Instrument DP3003 digital DC power supply. The electrolyte with the maximum conductivity was placed in between two ions-blocking stainless steel (SS) electrodes where mounted in a Teflon case. The polarization process starts at 0.80 V working potential at room temperature. Both ion (*t_ionic_*) and electron (*t_elec_*) transfer numbers were calculated using the following equation:

(1) $$t_{ionic}=\frac{I_{b}-I_{s}}{I_{b}}$$

(2) $$t_{elec}=1-t_{ionic}$$

where; *I_b_* is the onset current and *I_s_* is the steady state current.

#### 2.3.3. Linear Sweep Voltammetry (LSV)

The electrolyte potential window was examined with linear sweep voltammetry (LSV). Similar to TNM measurements, the cell arrangement was organized. The potentiostat Digi-IVY DY2300 was arranged for the measurement at 20 mV/s scan rate. The potential range between 0 and 3.5 V was chosen at ambient temperature.

#### 2.3.4. Cyclic Voltammetry (CV) of the EDLC

The preliminary test for the constructed EDLC was done at room temperature using cyclic voltammetry (CV) to determine the energy storage mechanism. Digi-IVY DY2300 potentiostat was used at various scan rates of 10, 20, 50, and 100 mV/s.

## 3. Results and Discussion

### 3.1. XRD Analysis

**Figure 1:**
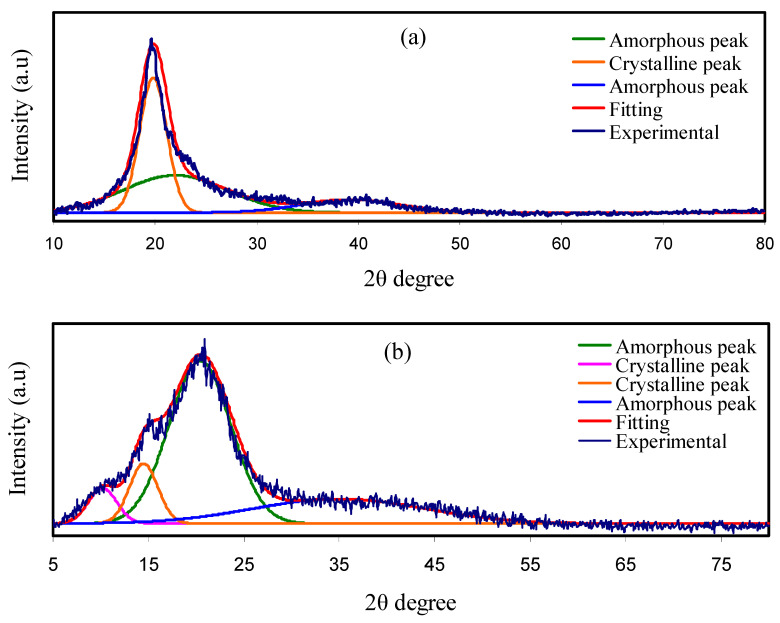

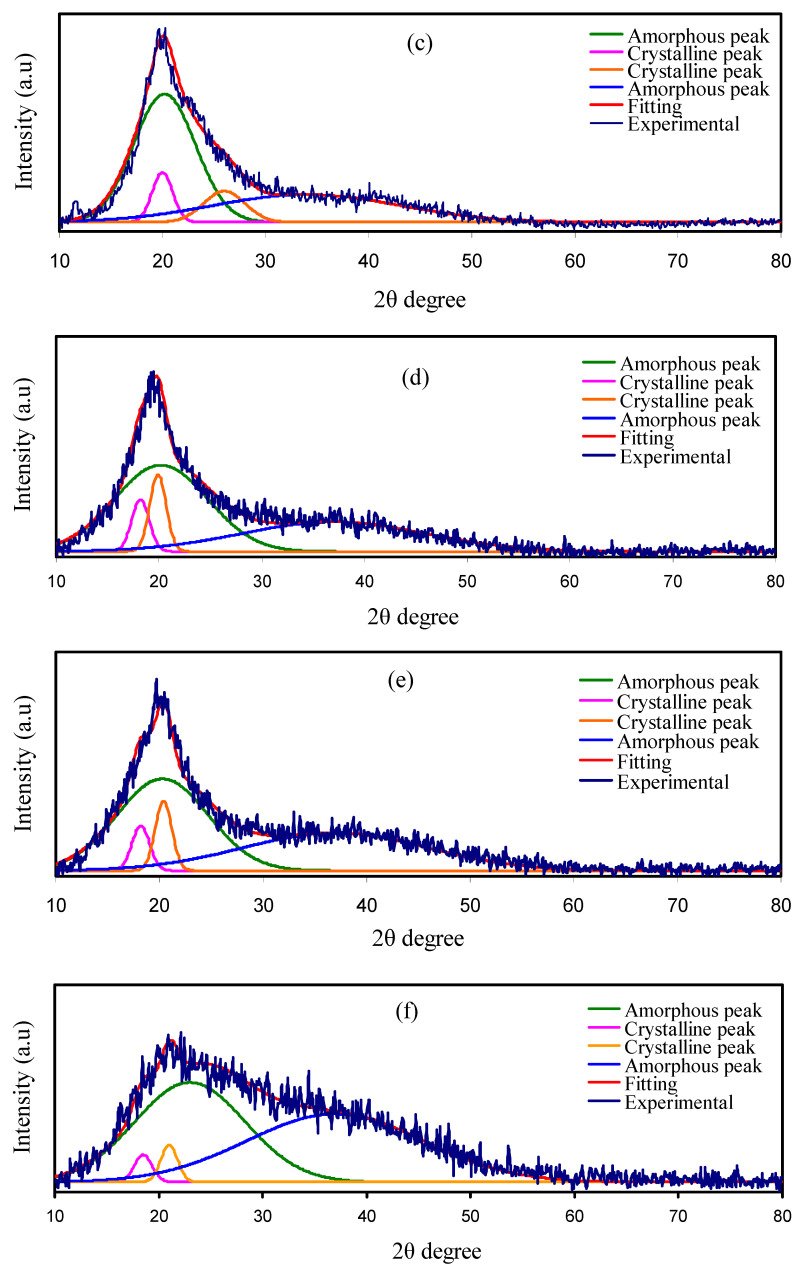
XRD pattern for (**a**) pure PVA, (**b**) pure CS, (**c**) CS:PVA blend, (**d**) CSPVMG1, (**e**) CSPVMG2, and (**f**) CSPVMG3.

The XRD pattern for the CS:PVA:Mg(CF_3_SO_3_)_2_ blend electrolytes doped with different glycerol amount is portrayed in Figure 1. The XRD spectra of pure PVA and CS films are indicated in Figure 1a,b. Previous study showed that the two distinct crystalline peaks at 2θ = 15.1° and 20.9° are feature of pure CS film. These crystalline peaks at 15.1° and 20.9° correspond to the reflection planes of (110) and (220), respectively. The broad peak at 2θ extended from 35° to 55° is the feature of the amorphous structure of CS [36]. A peak at around 19° proofs the semi-crystalline structure of pure PVA. The PVA attachment with OH groups along the main chain is sufficient to have a strong intramolecular and intermolecular hydrogen bonding. Remarkably, a broad peak centered at 2θ = 40.7° refers to an amorphous structure in PVA [37].

The deconvolution route is performed so as to specify the amorphous peaks and crystalline peaks [38]. The degree of crystallinity (*Xc*) of the peaks is obtained from the XRD deconvolution, as indicated in Figure 1. The broad and large peaks indicate the amorphous region, whereas the narrow, sharp, and small peaks denote crystalline peaks. This research indicated that as the 50 wt.% of CS was blended with 50 wt.% of PVA, the intensity of the diffraction peaks decreased and broadened (see Figure 1c). In addition, the crystalline peak of PVA at 2θ = 19°changed into two smaller diffraction peaks, as documented in literature [39,40]. This is caused by hydrogen bonding disruption owing to the dominance of amorphous structure in the blend system. Thus, polymer blending could be considered as an effective methodology to decrease the crystalline segment of PVA. It is notable to see that the *Xc* is reduced upon the addition of CS content (see Table 2). The *Xc* of CS in this research is quite close to the previous report [41]. As clear in Figure 1d–f, the crystalline peaks in CSPVMG1 become less sharp and smaller due to the inclusion of glycerol. Further glycerol inclusion up to 42 wt.% will cause to produce lesser crystalline peaks and as exhibited in CSPVMG3. The *Xc* for each system was achieved with Equation (4) and is shown in Table 2. The *Xc* of the CSPVMG1 system is 13.87. The lowest *Xc*, which is 4.55, is acquired for CSPVMG3. This shows that CSPVMG3 is the most amorphous system. The amorphous structure dominance accelerates polymer backbone segmental motion, which increases the ions transportation and conductivity [37].

The conductivity values follow the trend of degree of crystallinity.

(3) $$Xc=\frac{A_{C}}{A_{T}}\times 100\%$$

where *A_c_* and *A_T_* are the areas of total crystalline and the area of total amorphous and crystalline, respectively that is obtained using the deconvolution method with the OriginPro software. The Gaussian function mode was performed to fit the XRD spectra. Compared to the pure films and CS:PVA blend, the *Xc* in the plasticized systems is significantly decreased (see Table 2).

### 3.2. Dielectric Properties

To deal with the mechanism of ions transport in the electrolyte systems and the polarization effect at the sample/electrode interface, the dielectric properties were determined. The behavior of ion conduction inside the polymer electrolyte was examined. The two dielectric properties are dielectric constant and dielectric loss. Generally, the energy stored in each cycle, and polarity of the polymers were specified from the dielectric constant, whereas the waste energy of ions mobility represented by dielectric loss [42,43,44]. The dielectric behavior, such as dielectric constant (*ε*′), and dielectric loss (*ε*″) of the glycerolized CS:PVA:Mg(CF_3_SO_3_)_2_ electrolyte systems were determined by Equations (5) and (6):

(4) $$\varepsilon^{\prime }=\frac{Z^{\prime \prime }}{\omega C_{0}\left(Z^{\prime }{}^{2}+Z^{\prime \prime }{}^{2}\right)}$$

(5) $$\varepsilon^{\prime \prime }=\frac{Z^{\prime }}{\omega C_{0}\left(Z^{\prime }{}^{2}+Z^{\prime \prime }{}^{2}\right)}$$

where *ε*″ and *ε*′ refer to dielectric loss and dielectric constant, correspondingly. The *ω* stands for the applied field angular frequency (*ω* = 2*πf*).

**Figure 2:**
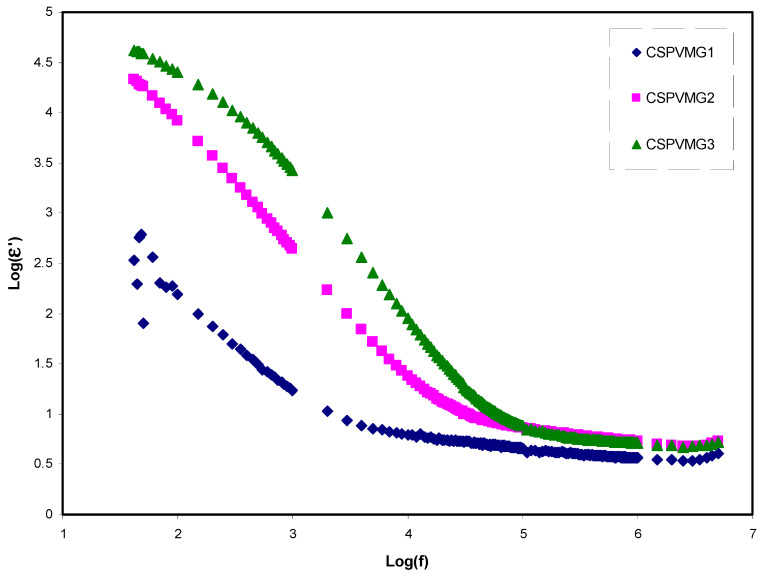
Variation of logarithmdielectric constant (*ε*′) versus frequency for the CSPVMG1, CSPVMG2, and CSPVMG3 electrolyte samples at ambient temperature.

**Figure 3:**
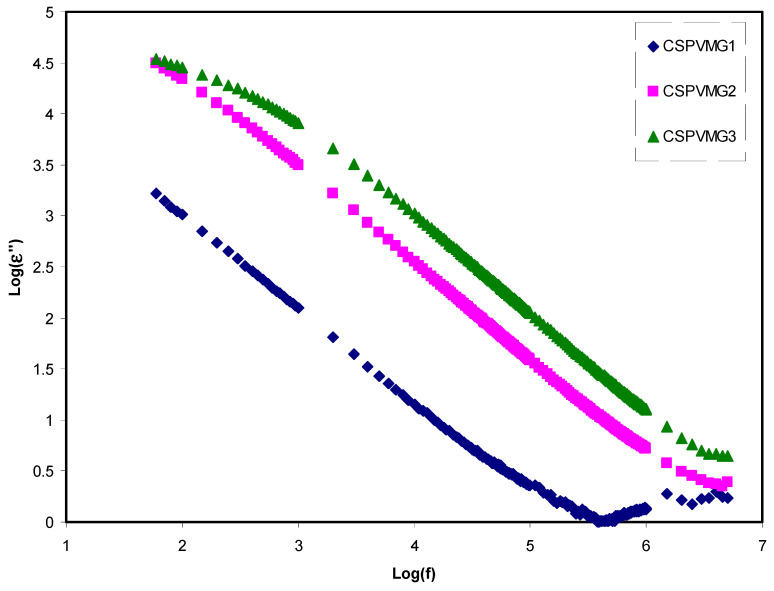
Variation of logarithm dielectric loss (*ε*″) versus frequency for the CSPVMG1, CSPVMG2, and CSPVMG3 electrolyte samples at ambient temperature.

The *C_o_* is the capacitance, which is equal to *ε_o_A*/*t*, where *ε_o_* refers the free space permittivity, *A* stands for the electrodes area, and *t* refers the sample thickness. Figure 2 and Figure 3 demonstrate the variation of dielectric parameters (i.e., *ε*′ and *ε*″) along with the frequency for the CSPVMG systems at ambient temperature. It is interesting to notice that the dielectric parameters are quite high at low (log) frequencies, indicating the existence of electrode polarization (EP) and space charge impacts [45,46,47]. The power of the frequency can easily be identified at intermediate frequency regions due to the dominance of the EP effect. A previous study established that the EP region is crucial for calculating the mobility and effective ion concentration [48]. Thus, the high value of EP means high carrier density. The existences of high charge carriers are important to produce EDLC devices with high capacitance. However, the dielectric value drops with rising frequency until it arrives constant values at high frequencies due to the periodic reversal of the electric field taken place quickly, reflecting no ion dispersion toward the field. In terms of energy storage, a buildup of charges at the electrodes and electrolytes interfaces results in the electric dispersion in the intermediate-frequency regions [49,50]. Based on Klein et al.’s [48] approach, the behavior of *ε*′ and *ε*″ versus log scale of frequency should plateau at a lower frequency and then a power law dispersion of *ε*′ and *ε*″ spectra must be identified at intermediate frequency regions. These phenomena can be seen in Figure 2 and Figure 3. These figures explain that the (*ε*′, *ε*″) values increase with the quantity of glycerol plasticizer into the system, and a sharp rise in the (*ε*′) for the CSPVMG3 electrolyte sample indicates the high ionic conductor at low-frequency [51].

**Figure 4:**
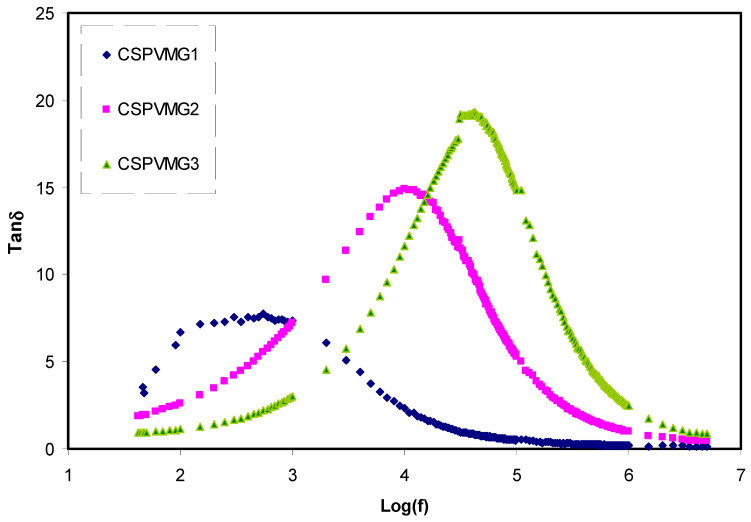
Variation of loss tangent (tanδ) versus frequency for the CSPVMG1, CSPVMG2, and CSPVMG3 electrolyte samples at room temperature.

To detect the relaxation behavior in the CS:PVA:Mg(CF_3_SO_3_)_2_ systems, the tangent loss (tanδ) was analyzed. Figure 4 provides insight into the variation of tangent loss (tanδ) along with the frequency for the CSPVMG films at ambient temperature. It is clear that the tangent loss (tanδ) raises with increasing frequency, and the maximum value at a definite frequency is observed and beyond it commences to decrease. This is resulted from faster increasing in ohmic component (i.e., active current) than its capacitive counterpart (i.e., reactive) at the lower frequency [52]. The cause for the decline in (tanδ) at a high-frequency region is attributed to independency of an ohmic component to the frequency and capacitive component rise in proportion to the frequency [53]. Furthermore, the presence of broad peaks at a mid-frequency designates the presence of the non-Debye type of relaxation process [54]. Consequently, the loss spectra peaks and their corresponding shifts with various quantity of glycerol plasticizer confirm the dielectric relaxation (DR) phenomena [55]. The DR occurs in materials subjected to an external electric field (EEF). When an EEF is applied to a medium such as polymer electrolyte, microscopic rearrangement (polarization) of the particles comprising the medium including electrons, atoms, molecules, and ions takes place so as to align with the external field. In general, a time lag develops between the EEF and the polarization of the polymer electrolyte. The relaxation time depends on the different characteristic rearrangement time of the medium. Previous studies revealed that plasticizer inclusion into the polymer electrolytes results in increasing the amorphous phase and dissociate more slats [56,57,58]. In plasticized polymer electrolytes chain and segmental motion increases [59,60]. It is reported that the segmental movement increment of polymer chains decreases the relaxation time as a result aids the mechanism of transportation. This is mathematically determined from τ = 1/2π*f*_max_, where τ refers to the relaxation time for the carrier species. This means that, as the ions mobility increases, the relaxation time is decreased, which shows the increment in conductivity of ions as a result of the rise in the system segmental movement [61].

### 3.3. Impedance Analysis

**Figure 5:**
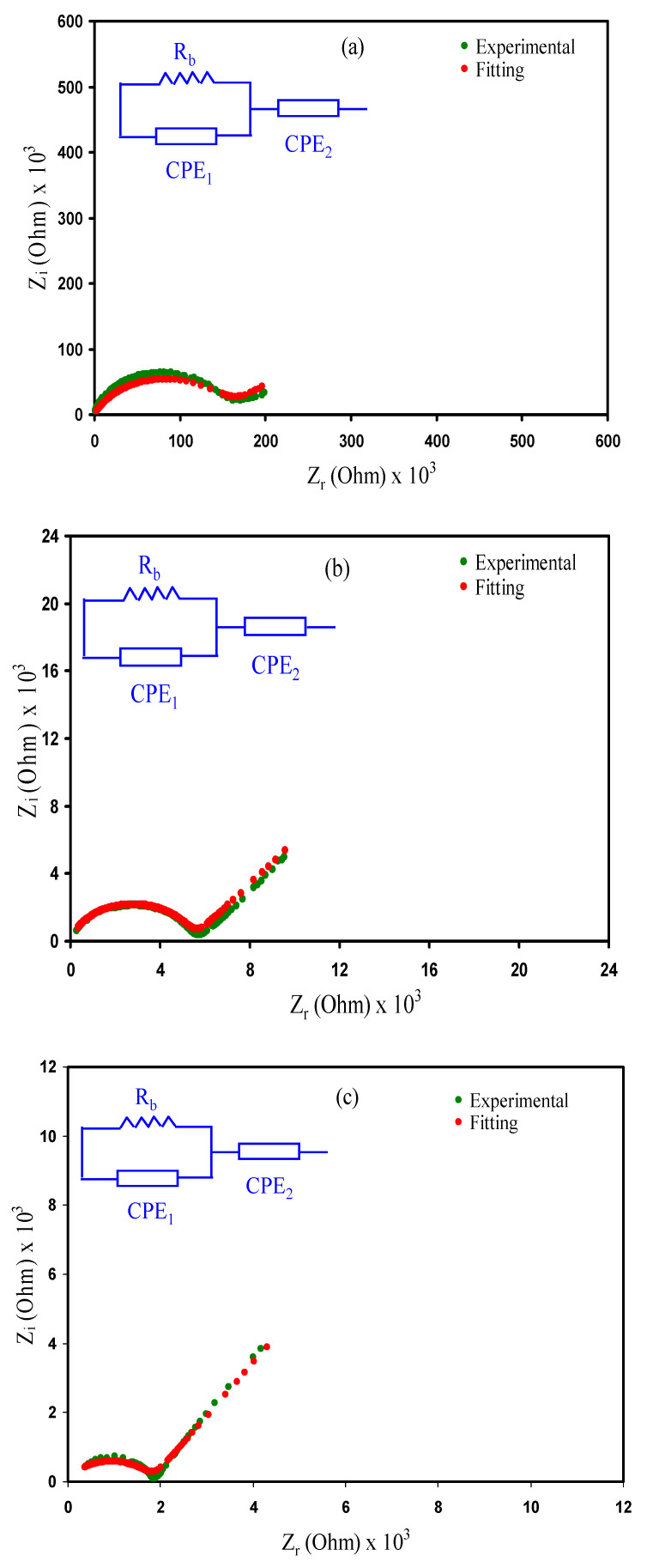
Complex impedance plot (*Z_i_* versus *Z_r_*) for the (**a**) CSPVMG1, (**b**) CSPVMG2, and (**c**) CSPVMG3 films at room temperature.

In present work, the SPE films containing a various quantity of glycerol plasticizer were characterized using the AC impedance spectroscopy technique. For this purpose, the Nyquist plots of impedance spectra (*Z_i_* versus *Z_r_*) for the all glycerolized CS: PVA: Mg(CF_3_SO_3_)_2_ systems at room temperature are exhibited in Figure 5a–c. All spectra are featured by low-frequencies spike (i.e., tail), and high-frequencies semicircle [62,63,64]. From the data analysis, it is achieved that incorporation of glycerol plasticizer into the CSPVMG based solid polymer electrolyte systems enhances the ionic conductivity. It is also observed that all the electrolyte samples exhibited an inclined spike with the semicircle, confirming their semiconducting characteristics [65]. The appearance of the semicircle at the high-frequencies is ascribed to the bulk character of the polymer electrolytes and the straight-line region at the low-frequencies is caused by blocking electrode effect [66]. It is seen that the diameter of half-circle diminishes with raising the quantity of glycerol. From the analysis, the CSPVMG films ionic conductivity (*σ_dc_*) has been calculated using the bulk resistance (*R_b_*) acquired from extrapolating the spike to the (*Zr-axis*) at the higher intercept of a semicircle, using the following equation:

(6) $$\sigma_{dc}=\left(\frac{1}{R_{b}}\right)\times \left(\frac{t}{A}\right)$$

where; *t* is the film thickness, and *A* is equal to the known electrode area. In the present work, the obtained maximum ionic conductivity is 1.016 × 10^−5^ S cm^−1^ when 42 w.t.% plasticizer (i.e., glycerol) was added at room temperature, as listed in Table 3. It is clear that the increment of the glycerol quantity to the system results in considerable improvement in the ionic conductivity, indicating an increase in the charge carrier mobility [67,68]. Inside the polymer body, (i.e., electrolyte films) there is an ionic crystal where columbic interaction is disrupted by glycerol plasticizer. Thereby, free ion and the film flexibility are dominating [59]. The present results have shown higher conductivity compared to our earlier work that the PVA: CS: NH_4_I system without plasticizer was studied that 2 × 10^−7^ S cm^−1^ was obtained [13]. However, in another study [69] CS:Starch:LiClO_4_:glycerol system was examined and a maximum ions conductivity of 3.7 × 10^−4^ S cm^−1^ at room temperature was obtained which is slightly higher than the current value of conductivity.

The electrical equivalent circuit (EEC) depiction is performed in impedance analysis as it offers the total picture of the systems [70]. The Cole-Cole plot for the systems comprises a semicircle arc and a tail. Thus, the EEC could be indicated by a connection of *R_b_* and constant phase element (CPE) in parallel and with another CPE in series [70]. The *Z_r_* and *Z_i_* values linked to the EEC are expressed as:

(7) $$Z_{CPE}=\frac{1}{C\omega^{p}}\left[\cos\left(\frac{\pi p}{2}\right)-i\sin\left(\frac{\pi p}{2}\right)\right]$$

(8) $$Z_{r}=\frac{R_{b}{}^{2}\left(A\right)+R_{b}}{2R_{b}\left(A\right)+B+1}+\frac{\cos\left(\frac{\pi p_{2}}{2}\right)}{C_{2}\omega^{p2}}$$

where

(9) $$A=C_{1}\omega^{p1}\cos\left(\frac{\pi p_{1}}{2}\right),B=R_{b}{}^{2}C_{1}{}^{2}\omega^{2p1}$$

(10) $$Z_{i}=\frac{R_{b}{}^{2}\left(C\right)}{2R_{b}\left(A\right)+B+1}+\frac{\sin\left(\frac{\pi p_{2}}{2}\right)}{C_{2}\omega^{p2}}$$

where

(11) $$C=C_{1}\omega^{p1}\sin\left(\frac{\pi p_{1}}{2}\right)$$

where *C*_1_ refers the capacitance at high frequency, *C*_2_ refers the capacitance at low frequency, *p*_1_ refers the departure of the circle from the imaginary axis, and *p*_2_ refers to the departure of the spike from the real axis. The parameters of the EEC for the systems are indicated in Table 4.

In Table 4, *K*_1_ and *K*_2_ refers the reciprocal of capacitance at high and low frequency, respectively.

### 3.4. Transfer Number Measurement (TNM) Study

**Figure 6:**
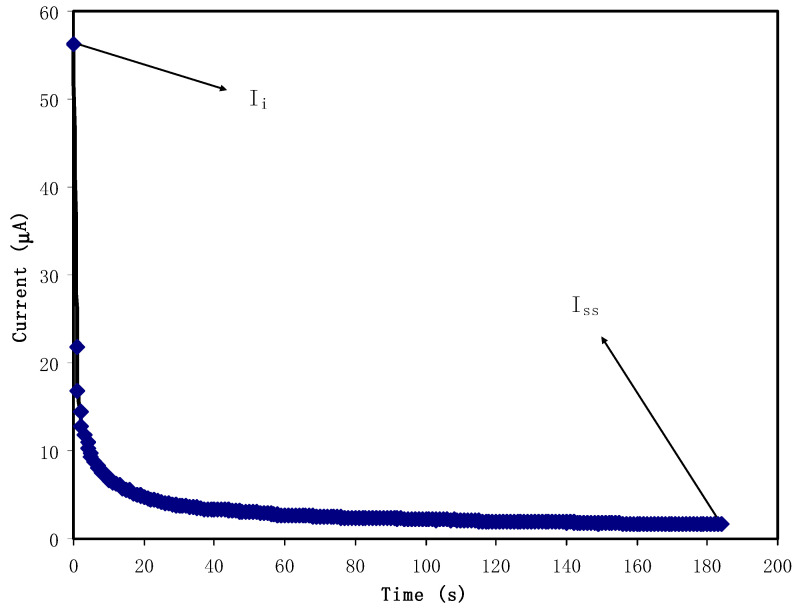
Polarization of SS| best conducting electrolyte | SS at 0.80 V.

There are two charge carrier species in a salt-doped electrolyte; electrons and ions. In construction of EDLC, it is vital to determine the identity of species which is the main charge carrier. Figure 6 shows the polarization of SS| high conducting electrolyte | SS at holding voltage of 0.80 V. It is seen that a huge current of 56.2 μA appears at beginning stage. It is known that both electrons and ions contribute in the whole conductivity process in the electrolyte; thus, providing a high value of *I_b_*. After 10 s, the current starts to drop to 6.9 μA. This lowering in current response resulted from surface blocking of the electrode (stainless steel) due to ion accumulation. Then, a steady state current is reached with a constant value of 1.6 μA. The accumulation of maximum number of ions at stainless steel electrode leads to increasing the polarization or a charge double-layer at SS | electrolyte interfacial region. As a result, at the steady-state stage, the current response is due to electrons rather than ions. The electrolyte under study (CSPVMG3) possesses *t_ionic_* and *t_elec_* of 0.972 and 0.028, respectively. The combined polyethlylene glycol (PEG) and magnesium acetate (Mg(CH_3_COO)_2_) system has shown *t_ionic_* of 0.97 as reported by Polu and Kumar [71]. In other study carried by Shanmuga et al. [72], it was confirmed that the dominancy of ions in a system of I-carrageenan: magnesim nitrate (Mg(NO_3_)_2_) with large *t_ionic_* of 0.97. In thee PVA-Mg(CH_3_COO)_2_ system, ions are the primary charge carriers by achieving *t_ionic_* of 0.96 [73].

To be implemented in EDLC, the electrolyte has to have high ionic transference number. Herein, more charge double-layer can be developed with high number of free ions where in CSPVMG3, it is confirmed that ions are the primary charge carriers.

### 3.5. Linear Sweep Voltammetry (LSV)

**Figure 7:**
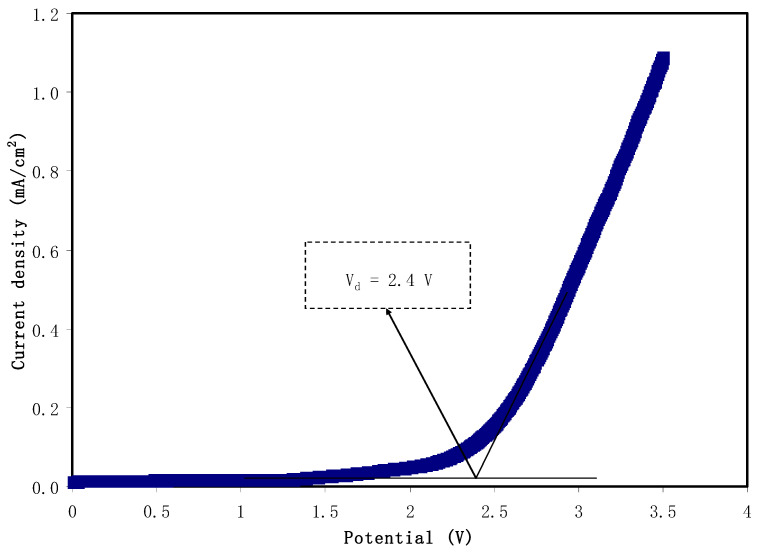
LSV plot for CSPVMG3 at a scan rate of 20 mV/s.

One of the key criteria of the utilization of electrolyte in energy devices is tolerance toward applied potential which is so called potential stability. Therefore, for an electrolyte to be used in the energy devices, it is necessary to have high electrochemical stability. This is to make sure that the electrolyte tolerates rapid charge-discharge process in the EDLC. Figure 7 shows the current-potential response in the potential window of 3.5 V. It is seen that there is no considerable change in current value as the potential reaches 2.4 V. Beyond 2.4 V, a sharp rise in current value is seen. This indicates oxidation of CSPVMG3 starts at 2.4 V; in other words, the decomposition voltage (*V_d_*) of CSPVMG3 is 2.4 V. Jo et al. [74] documented the potential stability window for a magnesium salt-based poly(ether urethane) electrolyte that is stable up to 1.9 V. Similarly, an electrolyte system of polymethyl methacrylate (PMMA) as the host polymer and magnesium triflate (Mg(CF_3_SO_3_)_2_) as the ionic source was studied by Zainol et al. [75]. This electrolyte system has shown a potential stability range from 0 to 2.4 V. In another study that carried out for pectin-magnesium chloride (MgCl_2_) system, an electrochemical stability of 2.05 V was recorded [76]. This magnesium-based electrolyte has almost similar results to the present study. Hamsan et al. [77,78] reported that the potential stability window for the electrolyte systems of CS: Mg(CH_3_COO)_2_: glycerol and CS: MgCl_2_: glycerol is stable up to 2.4 V and 1.83 V, respectively and the authors used both of the electrolytes in EDLC application. Thus, CSPVMG3 can be used as the electrodes separator (i.e., electrolyte medium) in the construction of the EDLC.

### 3.6. Cyclic Voltammetry (CV) 

**Figure 8:**
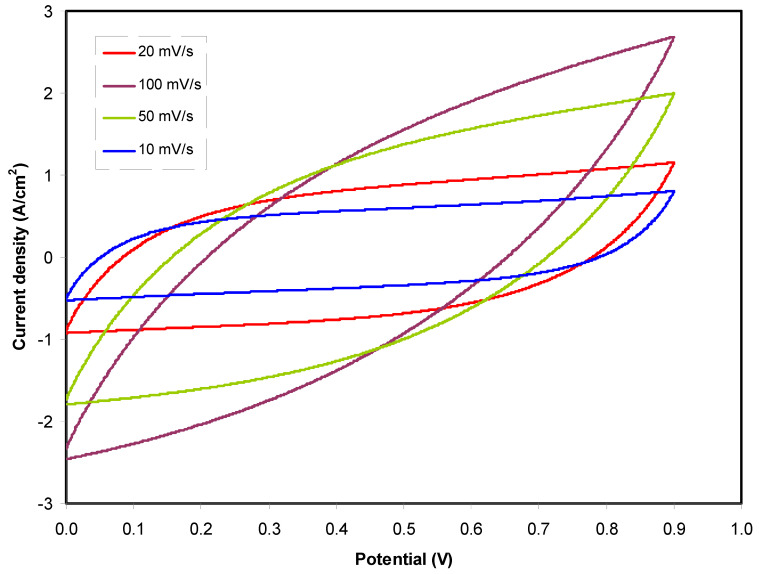
CV plot of the constructed EDLC at different scan rates from 0 V to 0.9 V.

In the characterization process of EDLC, cyclic voltammetry (CV) was recorded. The effect of scan rate on CV shape is shown in Figure 8. In all scan rate values, the CV responses are featured by leaf shape. It is well-known that a perfect capacitor possesses perfect rectangular shape in the CV response. However, in a real capacitor, several factors have to be taken into consideration, such as porosity and internal resistance of the electrode. These two factors impact the relationship between current and voltage [79]. From the recorded CV response of the constructed EDLC, it is seen that there is no reduction/oxidation peak. From the CV response, the mechanism of energy storage of an EDLC is verified where non-Faradaic process occurs. In this process, cations and anions undergo adsorption and de-sorption processes at negative and positive electrode, respectively, other than intercalation/deintercalation process [80]. The specific capacitance (*C_cyc_*) was determined from CV using the equation shown below [9]:

(12) $$C_{cyc}=\int_{V_{1}}^{V_{2}}\frac{I\left(V\right)dV}{2mS\left(V_{2}-V_{1}\right)}$$

where *ʃI*(*V*)*dV* is the area of CV profile, which was calculated using Origin 9.0 software. In this work, the CV was run between *V*_1_ (0 V) and *V*_2_ (0.9 V), *m* and *S* are the mass of active material used and scan rate, correspondingly.

The calculated *C_cyc_* are listed in Table 5. It is clear that a high *C_cyc_* is recorded at lower scan rate while it is not the case at larger scan rates. In this work, ions create a stable double-layer charge at the interfacial region between the AC electrode surfaces at low scan rates. Under this condition, a large value of capacitance is achieved. At the low scan rate, almost perfect plateau region can be observed, indicating that the free ions migrate at a roughly constant rate and therefore create the buildup of ions at the electrodes and electrolytes interfaces with small ohmic resistance. It is important to note that, at the low scan rate, a thick diffuse layer will form at the interface region between electrolyte and electrode. Meanwhile, at the high scan rate, a thin diffuse layer leads to a faster ionic conduction which hinders the formation of the desired polarization process [81].

Compared to our previous works in which MgCl_2_ [78] and MgCH_3_COO [77] were used as Mg^2+^ ion providers; the *C_cyc_* of the present work is lower. Based on our previous work [77], EDLC with CS:Mg(CH_3_COO)_2_:glycerol possesses specific capacitance of 39.72 F/g at the 10 mV/s. Moreover, the EDLC with CS:MgCl_2_:glycerol possesses specific capacitance of 50 F/g at the 10 mV/s [78]. In this work, Mg(CF_3_SO_3_)_2_ is used as the ionic source. Mg(CF_3_SO_3_)has lattice energy (*U_L_*) of 1967.51 kJ/moL, which is slightly lower than those estimated for Mg(CH_3_COO)_2_ (2627.64 kJ/moL) and MgCl_2_ (2582.13). The *U_L_* for Mg(CF_3_SO_3_)_2_, Mg(NO_3_)_2_, MgCl_2_,and Mg(CH_3_COO)_2_ were calculated using Kapustinskii’s equation (see Table 6) [82,83]:

(13) $$U_{L}=\frac{1202\left(v\right)\left(Z^{+}\right)\left(Z^{-}\right)}{d_{o}}\left(1-\frac{0.345}{d_{o}}\right)$$

where *v* is the ion number and *d_o_* is the sum of cation species radius and anion species radius. *Z^+^* and *Z^−^* are the number of charges. It was well reported [84,85] that salts with lower *U_L_* tends to associate more easily compared to salts with higher *U_L_*. Due to the lower value of *U_L_*, there is a greater chance for the cation and anion to associate again. Previous studies [84,85], revealed that polymer electrolytes impregnated with low lattice energy salts such as NH_4_BF_4_ results in high association of ions and the leakage of a large amount of aggregate salts to the surface of the films and lower DC conductivities (10^−7^–10^−8^ S/cm). Consequently, few free ions are available, thus resulting in a lower capacitance value. This could be due to the ionic radius of the anion species. (CF_3_SO_3_)^−^ anion has ionic radius of 256 pm, which is bigger than CH_3_COO^−^ anion (162 pm) and Cl^−^ anion (167 pm). Bigger ions have lower mobility compared to small ions as it is harder for the bigger ions to travel from the electrolyte to the surface of the electrode. This could be the reason of lower capacitance value of the EDLC device with CS:PVA:Mg(CF_3_SO_3_)_2_):glycerol in this work. Table 6 shows the lattice energy and anion size for some magnesium salts. Table 7 shows a comparison of the present work with other reported ones using carbon electrode materials based on EDLC in terms of specific capacitance value obtained from CV response.

## 4. Conclusions

The preparation of CS: PVA: Mg(CF_3_SO_3_)_2_:glycerol polymer electrolytes was performed via a solution cast methodology. Chitosan and PVA were used as the polymer blend host where ions provided by magnesium triflate (Mg(CF_3_SO_3_)_2_) that participates in the conduction process. The insertion of 42 wt.% glycerol improved the conductivity to 1.016 × 10^−^^5^ S cm^−1^. The outcome from the XRD examination displayed that the maximum conducting plasticized system possesses the smallest *Xc* which was determined to be 4.55. The dielectric relaxation analysis has displayed that the peak emergence obeys the non-Debye kind of relaxation process. It was concluded that ions in CS: PVA: Mg(CF_3_SO_3_)_2_:glycerol is the main charge carrier, as evidenced by transference number analysis that *t_ionic_*(0.972) > *t_elec_*(0.028). Polymer electrolyte has shown a satisfactory tolerance towards an applied potential up to 2.4 V, indicating eligibility of the electrolyte in the fabrication of the EDLC. It is also proven that the electrolyte system is characteristically capacitive to a large extent. The effect of scan rate on specific capacitance is clarified where a low scan rate increases specific capacitance, and at a high scan rate, the effect is reversed. The role of the lattice energy of salt is important to be considered prior to fabricate EDLC devises. It appears that very low lattice energy magnesium salts are not favorable for EDLC device fabrication.

## Figures and Tables

**Table 1:** The designation and composition for the glycerol plasticized of PVA:CS:Mg(CF_3_SO_3_)_2_ systems.

Sample Designation	PVA (g)	CS (g)	Mg (CF_3_SO_3_)_2_ (g)	Glycerol (g)	Glycerol W.t.%
CSPVMG1	0.5	0.5	0.666	0.271	14
CSPVMG2	0.5	0.5	0.666	0.647	28
CSPVMG3	0.5	0.5	0.666	1.206	42

**Table 2:** The degree of crystallinity using XRD spectra deconvolution.

Sample Designation	Degree of Crystallinity (%)
Pure PVA	41.68
Pure CS	15.97
CS:PVA	15
CSPVMG1	13.87
CSPVMG2	11.26
CSPVMG3	4.55

**Table 3:** Ionic conductivity of glycerol plasticized of CS:PVA:Mg(CF_3_SO_3_)_2_ systems at room temperature.

Sample Designation	*R_b_*	*σ_dc_* (S cm^−1^)
CSPVMG1	1.5 × 10^5^	1.286 × 10^−7^
CSPVMG2	5.6 × 10^3^	3.446 × 10^−6^
CSPVMG3	1.9 × 10^3^	1.016 × 10^−5^

**Table 4:** The EEC fitting parameters for CPEs system at room temperature.

Sample Designation	*K*_1_ (F^−1^)	*K*_2_ (F^−1^)	*C*_1_ (F)	*C*_2_ (F)
CSPVMG1	6.0 × 10^8^	8.0 × 10^5^	1.67 × 10^−9^	1.25 × 10^−6^
CSPVMG2	5.0 × 10^8^	1.9 × 10^5^	2.0 × 10^−9^	5.26 × 10^−6^
CSPVMG3	5.0 × 10^7^	1.8 × 10^5^	2.0 × 10^−8^	5.56 × 10^−6^

**Table 5:** Calculated specific capacitance from the CV plot at different scan rates.

Scan rate (mV/s)	Area	*M* (g)	*V*_2_ − *V*_1_ (V)	*C_cyc_* (F/g)
100	3.24 × 10^−3^	2.43 × 10^−3^	0.9	7.41
50	3.13 × 10^−3^	2.43 × 10^−3^	0.9	14.31
20	2.21 × 10^−3^	2.43 × 10^−3^	0.9	25.26
10	1.43 × 10^−3^	2.43 × 10^−3^	0.9	32.69

**Table 6:** Lattice energy for some of magnesium salts.

Ammonium Salts	Cation	Anion	Cation Radius (pm) [83]	Anion Radius (pm) [83]	Lattice Energy (KJ/moL)
Mg(CF_3_SO_3_)_2_	Mg^+2^	CF_3_SO_3_^−^	72	256 [70]	1967.51
Mg(NO_3_)_2_	Mg^+2^	NO_3_^−^	72	185	2429.51
MgCl_2_	Mg^+2^	Cl^−^	72	167	2582.13
Mg(CH_3_COO)_2_	Mg^+2^	CH_3_COO^−^	72	162	2627.64

**Table 7:** Various reported carbon electrode materials based EDLC studies with their respective value of specific capacitance from CV.

System	C_cyc_(F/g)	Scan Rate (mV/s)	Reference
PVA-CH_3_COONH_4_	0.14	10	[85]
Starch-LiClO_4_	8.70	10	[86]
HEC-Mg(CF_3_SO_3_)_2_	21.40	5	[87]
EC-DMC-LiTFSI	24.00	5	[88]
P(VdF-HFP)-EMI-BTI	29.60	3	[89]
PEMA-Mg(CF_3_SO_3_)_2_	1.99	10	[90]
PVA-CS-NH_4_SCN-glycerol	25.05	10	[91]
PEMA-Mg(CF_3_SO_3_)_2_-BmImBr	7.34	10	[92]
PVA-NaCF_3_SO_3_	14.78	3	[93]
CS-PVA-Mg(CF_3_SO_3_)_2_:glycerol	32.69	10	This work
